# Career awareness as a mediator between communication skills and entrepreneurial tendencies in prospective sport managers

**DOI:** 10.1371/journal.pone.0358456

**Published:** 2026-09-15

**Authors:** Zafer Gayretli, Zülbiye Kaçay, Samet Zengin, Nuh Osman Yıldız, Laurentiu-Gabriel Talaghir, Teodora Mihaela Iconomescu, Gabriel Marian Manolache, Dumitru Marius Cosoreanu

**Affiliations:** 1 Karadeniz Technical University, Department of Physical Education, Trabzon, Türkiye; 2 Çanakkale Onsekiz Mart University, Faculty of Sport Sciences, Çanakkale, Türkiye; 3 Trabzon University, Faculty of Sport Sciences, Trabzon, Türkiye; 4 Bolu Abant İzzet Baysal University, Faculty of Sport Sciences, Bolu, Türkiye; 5 Dunarea de Jos University of Galati, Faculty of Physical Education and Sport, Galati, Romania; Universidade Europeia, Lisboa, PORTUGAL

## Abstract

Universities are increasingly expected to develop transferable competencies that improve graduates’ employability, yet little is known about the psychological mechanisms through which such competencies translate into entrepreneurial outcomes in sport management education. Drawing on social cognitive career theory (SCCT), this study examined whether career awareness mediates the relationship between communication skills and entrepreneurial tendencies among prospective sport managers. Cross-sectional data were collected from 622 undergraduate sport management students in Türkiye (280 women, 342 men) using the Communication Skills Scale, the Career Awareness Scale, and the Entrepreneurship Scale for University Students. Following a pre-specified mediation protocol, a pooled measurement model containing all constructs was evaluated by confirmatory factor analysis before the structural model was estimated by maximum likelihood; indirect effects were tested with bias-corrected bootstrapping based on 5,000 resamples. All hypothesised paths were supported. Communication skills predicted career awareness (β = 0.73, p < 0.001) and entrepreneurial tendencies (β = 0.50, p < 0.001), and career awareness predicted entrepreneurial tendencies (β = 0.45, p < 0.001). Career awareness partially mediated the relationship between communication skills and entrepreneurial tendencies (indirect effect = 0.33, 95% CI [0.25, 0.42]), transmitting approximately 40% of a total effect of 0.83. Together, the two predictors explained 79% of the variance in entrepreneurial tendencies. The findings extend SCCT to the sport management education context by identifying career awareness as a cognitive-developmental mechanism that converts interpersonal competence into entrepreneurial orientation, rather than treating communication as a direct antecedent. Practically, they indicate that communication training yields limited entrepreneurial returns unless it is deliberately coupled with career-awareness interventions: sport management programmes should embed structured career guidance, industry mentoring, and venture-based project work into applied communication coursework, and accreditation frameworks should treat career awareness as an assessable programme outcome.

## Introduction

Graduate employability has become a pressing concern in higher education systems worldwide, and it is particularly acute in Türkiye, where the expansion of undergraduate provision has outpaced the labour market’s capacity to absorb graduates into field-relevant positions. The difficulty is not primarily a shortage of qualifications but a mismatch between the competencies that curricula certify and those that employers reward. Where that mismatch persists, it depresses the returns to higher education, contributes to youth unemployment and weakens the contribution of universities to economic development. The policy response has been to reorient curricula towards competency-based and labour-market-responsive designs — but doing so requires knowing which competencies matter and, more importantly, how they come to matter.

On the first question there is considerable convergence. The Partnership for 21st Century Learning framework, the Assessment and Teaching of 21st Century Skills project and the OECD’s competency classification differ in their taxonomies, yet all three place communication, collaboration, initiative and career self-management among the transferable capabilities that contemporary work demands [1–3]. On the second question these frameworks are largely silent. They specify what graduates should be able to do, but not the process through which a capability developed in a classroom becomes an employment-relevant disposition. That translation process, rather than the inventory of competencies itself, is what curriculum designers need to understand.

Sport management is an instructive setting in which to examine it. The number of sport management programmes in Türkiye has grown rapidly, intensifying competition among graduates for a limited pool of formal managerial appointments. At the same time, much of the sector’s employment growth is occurring outside conventional posts — in event organisation, fitness ventures, athlete representation, sport marketing and consultancy. Employability in this field therefore depends not only on being hired but on being able to generate work. This makes entrepreneurial tendency a component of employability rather than an alternative to it. Yet the available evidence indicates that prospective sport managers approach graduation with pronounced occupational uncertainty and job-finding anxiety [4,5], and that their programmes often leave career pathways insufficiently articulated.

Two competencies recur in research on this population. Communication skill is treated as a foundational resource in a sector organised around sustained interaction with athletes, coaches, staff, sponsors and publics, and has been shown to be associated with entrepreneurial orientation among sport students [6]. Career awareness — students’ understanding of the pathways, requirements and opportunities that characterise their occupational field — has been examined as an outcome of educational provision and as a correlate of career self-efficacy [7,8]. Both literatures are informative, but both are overwhelmingly bivariate.

Three limitations follow, and together they define the gap this study addresses. First, the communication–entrepreneurship association has been documented as a direct effect, which leaves the intervening process unexamined; the relationship remains, in effect, a black box, and interventions built on it must proceed on the untested assumption that improving communication is sufficient. Second, although social cognitive career theory (SCCT) holds that personal competencies shape career outcomes through cognitive appraisals of the self and of the occupational world rather than directly [9,10], the theory is typically invoked in sport management education as a descriptive backdrop, with its mediating machinery neither specified nor tested. Third, career awareness has been studied as a dependent variable but not as the mechanism that converts interpersonal competence into entrepreneurial disposition — even though SCCT gives clear grounds for expecting it to serve that function.

These limitations yield the starting question that motivates the present study [11]: why do students with comparable communication skills differ so markedly in their entrepreneurial tendencies? Our contention is that the difference resides not in the skill itself but in whether students possess a structured understanding of the occupational field in which that skill could be deployed. Communication competence generates career-relevant learning experiences — conversations with practitioners, internship interactions, negotiation with stakeholders — and it is through these experiences that students acquire a clearer picture of career pathways and requirements. That clarified picture, in turn, is what makes entrepreneurial action appear feasible rather than merely desirable. On this account career awareness is not a parallel outcome of education but the mechanism linking competence to entrepreneurial disposition.

The present study therefore examines whether career awareness mediates the relationship between communication skills and entrepreneurial tendencies among prospective sport managers (i.e., undergraduate students enrolled in sport management programmes; the two terms are used interchangeably hereafter). In doing so it makes three contributions. Theoretically, it moves SCCT in sport management education from a descriptive framing to a testable mediational specification, and identifies which of the theory’s cognitive constructs carries the effect. Empirically, it estimates the proportion of the communication–entrepreneurship relationship that operates through career awareness, using a pre-specified mediation protocol on a large sample and a fully scripted, reproducible analysis. Practically, it establishes whether communication training alone is sufficient to raise entrepreneurial tendencies, or whether it must be paired with career-awareness provision to be effective — a distinction with direct consequences for curriculum design. The theoretical rationale for each hypothesised path is developed in the following section.

## Theoretical background and hypothesis development

### Social cognitive career theory as an organising framework

Social cognitive career theory explains occupational development as the product of reciprocal interaction among personal attributes, environmental conditions and overt behaviour [9,10]. Its central claim, and the reason it is well suited to the present problem, is that competencies do not act on career outcomes directly. They operate through learning experiences that furnish the individual with information about the self and about the world of work, and it is the cognitive appraisals formed from that information — self-efficacy beliefs, outcome expectations and occupational knowledge — that shape interests, goals and choice actions. Person inputs and contextual affordances such as institutional support, opportunity structures and socialisation patterns condition how readily those learning experiences accumulate.

This architecture has a methodological implication that is often overlooked in applied work. If competencies act through appraisals, then a model that regresses a career outcome directly on a competency is misspecified in a theoretically consequential way: it will recover an association while remaining silent on the process that produces it, and it cannot distinguish a competency that matters in its own right from one that matters only because of what it enables students to learn. Testing SCCT therefore requires an explicitly mediational specification. The present study adopts one, treating communication skill as a personal competence that generates career-relevant learning experiences, career awareness as the cognitive appraisal formed from those experiences, and entrepreneurial tendency as the resulting proactive career disposition.

### Communication skills and career awareness

Communication skill is widely recognised as the capacity to convey ideas intelligibly, to represent oneself accurately, to listen actively and to sustain interaction across differences of role and status [12]. It is a competence with unusual reach: because it is exercised in nearly every interpersonal encounter, differences in communicative facility compound over time into substantial differences in the quality and quantity of information an individual is able to obtain from others. Evidence that communicative capacity predicts broader socio-emotional competence is consistent with this cumulative view [13].

Career awareness refers to an individual’s structured understanding of occupational pathways, entry requirements, working conditions and development opportunities within a field, together with the perceived confidence to act on that understanding [7]. It is best understood as a precursor to other components of career development rather than a product of them: interest in an occupation is not sufficient for progress if the individual cannot describe how that occupation is entered or what it demands [14]. It is also unevenly distributed, since students frequently remain poorly informed about the pathways available to them, and it accrues gradually through sustained exposure rather than through single interventions [15].

The link between the two follows from how career awareness is acquired. Formal curricula supply only part of it; the remainder is obtained through interaction — questioning practitioners, interpreting what is said in internships, sustaining contact with alumni and supervisors, and asking follow-up questions when an answer is incomplete. Each of these is a communicative act, and each is performed better by students who can initiate conversation with unfamiliar interlocutors, express uncertainty without embarrassment and listen for detail. Communicatively skilled students consequently extract more career-relevant information from the same institutional environment than their less skilled peers, and they extract it earlier. In sport management the effect should be amplified, because the field’s employment structures are comparatively informal and much of what a student needs to know about them is not documented anywhere but is held tacitly by practitioners and transmitted conversationally [16,17]. In SCCT terms, communication skill functions as an enabling condition for the learning experiences from which occupational knowledge is constructed.

## H1: Communication skills positively predict career awareness

### Career awareness and entrepreneurial tendencies

Entrepreneurship denotes the creation of economic or social value through the recognition of opportunity and the assumption of financial, social and psychological risk in pursuit of it [18,19]. Entrepreneurial tendency, the construct examined here, is the corresponding individual disposition — a configuration of risk tolerance, innovativeness, opportunity recognition, self-efficacy, creativity and leadership orientation that predisposes a person towards such activity. In sport, this disposition has acquired practical weight as value creation has migrated towards event organisation, fitness ventures, digital sport services and consultancy, and as sport organisations have come to depend on managers who can initiate rather than merely administer [20–22].

Career awareness should bear on this disposition because opportunity recognition is knowledge-dependent. An opportunity is not a feature of the environment that any observer would register; it is a perceived gap between what a field currently provides and what it could provide, and perceiving such a gap presupposes a reasonably detailed map of the field’s existing provision. A student who cannot describe how sport organisations are structured, who they serve or where their services fall short has no basis on which to identify an unmet need, however risk-tolerant or creative that student may be. Career awareness supplies that map. It also affects the perceived feasibility of acting: knowing that self-initiated pathways exist in a field, and that they are populated by identifiable people who followed them, converts entrepreneurial action from an abstract possibility into a concrete option. Studies reporting associations between career awareness and career self-efficacy [8,23], and between career planning orientation and entrepreneurial orientation [24–26], are consistent with both mechanisms. In SCCT terms, career awareness contributes to the outcome expectations and world-of-work knowledge from which career goals and choice actions are formed.

## H2: Career awareness positively predicts entrepreneurial tendencies

### Communication skills and entrepreneurial tendencies

Communication skill should also bear on entrepreneurial tendency independently of what it teaches students about their field, because entrepreneurial activity is itself communicatively constituted. Assembling resources requires persuading their holders; building a venture requires recruiting collaborators; sustaining one requires negotiating with suppliers, sponsors and clients and representing the enterprise to audiences that are initially indifferent to it. Students who are confident in these activities may reasonably anticipate succeeding at them, and that anticipation is a component of entrepreneurial disposition rather than a consequence of it. Consistent with this, communication skills have been found to account for a substantial share of variance in entrepreneurial orientation among sport management students [6], and interventions targeting communication and leadership have been shown to raise professional competence in adjacent training contexts [27,28]. Research on entrepreneurship education among sport majors likewise identifies interpersonal capability as a recurring correlate of entrepreneurial psychology [29,30]. A direct path is therefore expected alongside the indirect one, which implies that career awareness should be modelled as a partial rather than a complete mediator.

## H3: Communication skills positively predict entrepreneurial tendencies when career awareness is controlled

### Career awareness as a mediating mechanism

Taken together, H1 to H3 describe a mediational process rather than three separate associations. Communication skill enlarges the career-relevant information a student can obtain from the educational environment; that information consolidates into an articulated understanding of occupational pathways and requirements; and that understanding is what renders entrepreneurial opportunities visible and entrepreneurial action feasible. The prediction is therefore not merely that all three paths are positive, but that a determinate share of the communication–entrepreneurship relationship is transmitted through career awareness — a quantity that only a mediational model can estimate and that carries the study’s principal practical implication, since it indicates how much of the return to communication training depends on career-awareness provision being in place.

Two considerations support the plausibility of this specification. First, communication has already been shown to operate as a mediating mechanism in sport settings, linking psychosocial resources to performance outcomes [31], which establishes that competencies of this kind transmit effects rather than only producing them. Second, career-related cognitions have been found to mediate between individual attributes and entrepreneurial outcomes in student populations outside sport [32,33], suggesting that the process proposed here is not specific to this field. Because a direct effect is also anticipated (H3), partial rather than full mediation is predicted.

## H4: Career awareness mediates the relationship between communication skills and entrepreneurial tendencies

**Fig 1:**
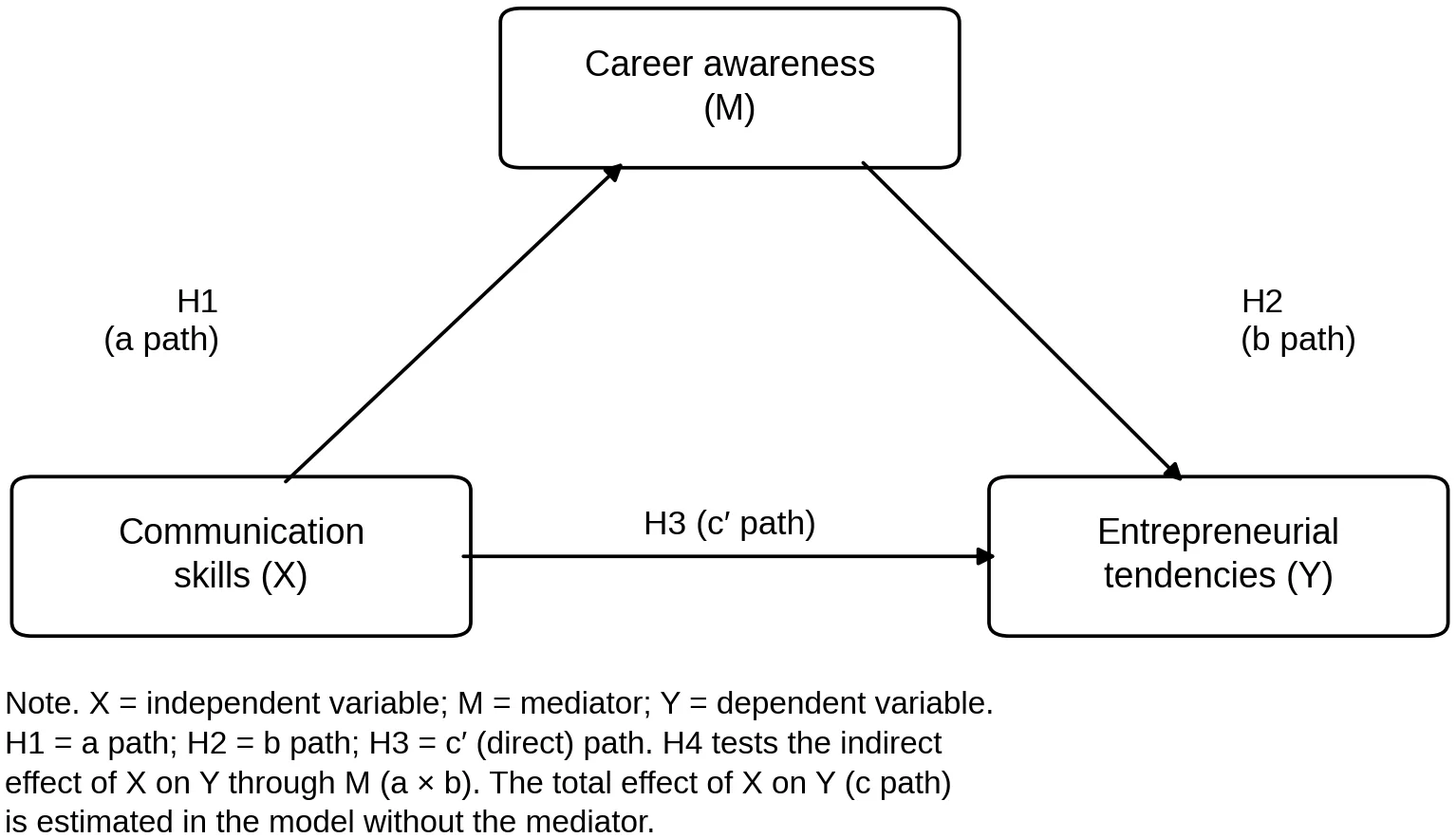
Conceptual model of the mediating role of career awareness. X = independent variable; M = mediator; Y = dependent variable. H1 = a path; H2 = b path; H3 = c′ (direct) path. H4 tests the indirect effect of X on Y through M (a × b). The total effect of X on Y (c path) is estimated in the model without the mediator.

The hypothesised model is presented in Fig 1.

## Materials and methods

### Study design and setting

The study employed a cross-sectional correlational design to examine associations among communication skills, career awareness and entrepreneurial tendencies in undergraduate sport management education [34]. Data were collected at a single time point from students enrolled in sport management programmes at Turkish public universities during the 2025–2026 academic year. The hypothesised model, together with the rationale for specifying career awareness as a mediator rather than a parallel outcome, is set out in the preceding section; the present section reports how that model was operationalised and tested.

### Population and sampling

The target population comprised undergraduate students enrolled in sport management (*spor yöneticiliği*) programmes at universities in Türkiye. According to Higher Education Council statistics, such programmes were offered at 89 universities and admitted 4,300 students in the 2025 admission cycle [35]. Total enrolment across all four years of study is not published at programme level and can therefore only be approximated: assuming a stable annual intake and no attrition, the accessible population is of the order of 17,200 students, against which the realised sample of 622 represents approximately 3.6%. This figure should be read as an order-of-magnitude estimate rather than a precise proportion, since it rests on assumptions about intake stability and progression that published statistics do not allow us to verify. Students from 16 of the 89 institutions (18.0%) are represented in the sample. We report these figures so that readers may judge the adequacy and representativeness of the sample directly rather than take it on trust.

Participants were recruited using non-probability criterion sampling. This strategy was adopted because no centralised sampling frame of individually identifiable sport management students exists in Türkiye: enrolment records are held separately by each institution and are not released for research purposes, which precludes probability sampling of the population as a whole. Criterion sampling was appropriate to the study objectives for two further reasons. First, the research question concerns a theoretically defined process — whether career awareness transmits the effect of communication skills — rather than the estimation of population parameters such as prevalence or mean levels, and the internal validity of a mediational test does not depend on the sample being probabilistically representative. Second, the construct of interest presupposes exposure to sport management curricula, so restricting eligibility to enrolled sport management students was substantively required rather than merely convenient. The single inclusion criterion was current enrolment in an undergraduate sport management programme; no exclusion criteria were applied.

Course instructors at sport management programmes were contacted by the research team and asked to circulate an electronic invitation containing the study information sheet and a link to the survey. Responses were received from students enrolled at 16 universities. The distribution was uneven, as is inherent to this recruitment method: the five largest contributing institutions accounted for 70.9% of the analytic sample (21.7%, 14.3%, 13.2%, 11.1% and 10.6% respectively), while five institutions contributed fewer than ten participants each. Because invitations were forwarded by instructors rather than issued directly by the research team, the number of students who received but did not open the invitation is unknown and a response rate cannot be calculated; this is noted as a limitation. Recruitment continued until the target sample size was reached, which accounts for the 625 responses initially obtained. Data were collected between 2 January and 15 March 2026 via an online survey platform.

### Sample size justification

Because the model was estimated as a latent-variable structural equation model with an indirect effect as the focal quantity, sample size adequacy was evaluated against three criteria rather than a single a priori regression calculation.

First, a sensitivity analysis indicated that the realised sample of 622 provides statistical power of 0.89 to detect an effect of f^2^ = 0.02 in a two-predictor regression framework at α = 0.05, and is sufficient to detect effects of f^2^ ≥ 0.016 with power of 0.80. The smallest effect estimated in the present model is substantially larger than this threshold, so the study was not underpowered for any hypothesised path.

Second, and more directly relevant, simulation evidence on required sample sizes for mediation indicates that a bias-corrected bootstrap test detects an indirect effect with medium-sized component paths (β ≈ 0.39) with 0.80 power at approximately N = 71, and that this test has the highest power among commonly used alternatives [36]. Both component paths estimated here exceed that magnitude, so the realised sample provides power well in excess of conventional requirements for the indirect effect.

Third, the structural model estimated 25 free parameters, giving a ratio of observations to estimated parameters of 24.9:1, well above the conventional minimum of 10:1 for maximum likelihood estimation [37].

### Measures

Data were collected using a four-part instrument. The first part recorded age, gender, year of study, self-reported academic achievement and city of residence. The remaining three parts comprised the measures described below.

All three instruments were adopted in their original form and original language; no translation, cross-cultural adaptation or item modification was undertaken, and consequently no adaptation procedures were required. This was possible because each scale was originally developed and validated in Turkish with Turkish undergraduate samples, and in the case of the career awareness measure specifically with sport sciences students. Permission to use the instruments was obtained from the original authors. Item wording, response format and scoring followed the original publications exactly, so the psychometric evidence reported by the scale developers applies to the present administration. Internal consistency was nevertheless re-estimated in the present sample, and construct validity was re-established through confirmatory factor analysis rather than assumed from the original studies.

#### Communication Skills Scale (CSS).

Interpersonal communication skills were assessed using the Communication Skills Scale developed by Korkut-Owen and Bugay with Turkish university students [12]. The scale comprises 25 items across four dimensions — communication principles and basic skills (10 items), self-expression (4 items), active listening and non-verbal communication (6 items), and willingness to communicate (5 items) — rated from 1 (never) to 5 (always), with no reverse-scored items. Higher scores indicate stronger communication skills. The developers reported a Cronbach’s alpha of 0.88 for the total scale. In the present sample, alpha was 0.918 for the total scale and ranged from 0.703 to 0.811 across dimensions.

#### Entrepreneurship Scale for University Students (ESUS).

Entrepreneurial tendencies were measured using the scale developed by Yılmaz and Sünbül with Turkish university students [38]. The scale comprises 36 items with a unidimensional structure, assessing entrepreneurship-related characteristics including risk-taking, innovativeness, opportunity recognition, self-efficacy, creativity and leadership, rated from 1 (never) to 5 (very often), with no reverse-scored items. Higher scores indicate stronger entrepreneurial tendencies. The developers established the single-factor structure through exploratory and confirmatory factor analysis and reported a total alpha of 0.90. In the present sample, alpha was 0.960.

#### Career Awareness Scale (CAS).

Career awareness was assessed using the Career Awareness Scale developed by Yaşar and Sunay specifically for students in sport sciences, which establishes its content relevance for the present population [7]. The scale comprises 18 items across four dimensions — professional development disposition (6 items), professional readiness (4 items), occupational awareness (4 items) and occupational self-confidence (4 items) — rated from 1 (strongly disagree) to 5 (strongly agree), with no reverse-scored items. Higher scores indicate greater career awareness. The developers confirmed the four-factor structure and reported a total alpha of 0.921, with subscale values between 0.79 and 0.83. In the present sample, alpha was 0.929 for the total scale and ranged from 0.782 to 0.834 across dimensions.

### Statistical protocol

Analyses followed a pre-specified six-stage protocol for testing mediation in a latent-variable framework, consistent with established methodological guidance on mediation analysis [39–43]. All analyses were conducted in Python 3.12 using maximum likelihood estimation as implemented in semopy 2.3.11 [44]. The complete analysis script is provided as S1 Script so that every value reported below can be reproduced exactly from the raw data.

#### Stage 1 — Data screening and assumption testing.

The survey instrument required a response to every item, so no data were missing. Univariate outliers were identified using standardised scores on the composite scale means, with cases outside ±3.29 excluded [45]; three cases met this criterion and were removed, yielding the analytic sample of 622. Multivariate outliers were assessed using Mahalanobis distance against a χ² criterion at p < 0.001. Univariate normality was evaluated using skewness and kurtosis, with values within ±3 taken as acceptable [46], and multivariate normality was assessed using Mardia’s coefficient of multivariate kurtosis [47]. Multicollinearity was examined using variance inflation factors (< 5), tolerance values (> 0.20) and the condition index (< 30).

#### Stage 2 — Common method variance.

Because all constructs were measured by self-report at a single time point, common method variance was assessed using Harman’s single-factor test, in which all 79 items were entered into an unrotated principal components analysis, and by comparing a single-factor confirmatory model in which all indicators loaded on one latent variable with the hypothesised measurement model [48].

#### Stage 3 — Measurement model.

Following the two-step approach [49], the measurement model was evaluated before the structural model. Confirmatory factor analysis was conducted first for each instrument separately at item level, and then as a single pooled model containing all constructs simultaneously, so that the model was assessed as an integrated whole rather than as a set of independent measurement models. In the pooled model, communication skills and career awareness were each represented by their four dimension scores and entrepreneurial tendencies by three item parcels of twelve items each, constructed by the item-to-construct balance method in which items are ranked by their mean inter-item correlation and allocated to parcels in serpentine order [50]; parcelling was used because the 79 individual items would otherwise yield an observation-to-parameter ratio below conventional minima at N = 622. Convergent validity was assessed using standardised factor loadings (≥ 0.50), average variance extracted (AVE ≥ 0.50) and composite reliability (CR ≥ 0.70). Discriminant validity was assessed in three complementary ways: the Fornell–Larcker criterion [51], the heterotrait–monotrait ratio of correlations with a 0.85 threshold [52], and nested model comparisons in which each latent correlation was constrained to unity.

#### Stage 4 — Structural model and causal steps.

The structural model was then estimated. For transparency and comparability with earlier work, the four causal steps of the classical framework are reported explicitly [39]: the total effect of communication skills on entrepreneurial tendencies (c path) estimated in a model without the mediator; the effect of communication skills on career awareness (a path); the effect of career awareness on entrepreneurial tendencies controlling for communication skills (b path); and the direct effect of communication skills on entrepreneurial tendencies controlling for career awareness (c′ path). We note, however, that contemporary treatments establish that a significant total effect is not a precondition for mediation and that the causal-steps procedure has comparatively low power [40–42,53]; accordingly, inference regarding mediation rests on the indirect effect itself rather than on the pattern of the causal steps.

#### Stage 5 — Indirect effect.

The indirect effect (a × b) was tested using bias-corrected bootstrapping with 5,000 resamples and 95% confidence intervals; a confidence interval excluding zero was taken as evidence of a statistically significant indirect effect [42]. Percentile intervals are reported alongside bias-corrected intervals, and the proportion of the total effect transmitted through the mediator is reported. Following current guidance, the descriptive labels “partial” and “complete” mediation are used only to characterise the observed pattern, and we note that this classification is sensitive to sample size and statistical power and does not itself constitute a substantive finding [42,54].

#### Stage 6 — Robustness.

Two robustness checks were specified in advance. First, because the design is cross-sectional and does not establish causal ordering, an alternative model reversing the positions of communication skills and career awareness was estimated and compared with the hypothesised model on fit. Second, the structural model was re-estimated with multivariate outliers excluded, to establish whether the conclusions depended on their retention. Model fit throughout was evaluated using the chi-square statistic with degrees of freedom, the chi-square/degrees of freedom ratio (χ²/df < 5), the comparative fit index and Tucker–Lewis index (both ≥ 0.90), and the root mean square error of approximation and standardised root mean square residual (both ≤ 0.08) [55]. Statistical significance was set at p < 0.05.

### Ethical approval and consent

The study was approved by the Trabzon University Social and Humanities Sciences Scientific Research and Publication Ethics Committee (7 November 2025; Decision No. 2025-11/1.10). Prospective participants were presented with an information sheet describing the purpose of the study, the voluntary nature of participation, the anonymity of responses and their right to withdraw before submission; written informed consent was obtained electronically before any items were displayed. Participation was voluntary and unpaid, responses were anonymous, and no identifying information was collected. The study involved no vulnerable groups and collected no sensitive personal data. All procedures conformed to the ethical principles of the Declaration of Helsinki and to relevant institutional and national research standards.

## Results

### Preliminary analyses

No data were missing. Three of the 625 initial cases exceeded the ± 3.29 standardised score criterion and were removed, leaving 622 cases for analysis. Skewness ranged from −0.258 to −0.031 and kurtosis from −0.522 to −0.074, all well within ±3 and consistent with approximate univariate normality (Table 3). Multicollinearity was not a concern: the variance inflation factor was 1.736, tolerance was 0.576 and the condition index was 2.176.

Mardia’s coefficient of multivariate kurtosis was 184.39 against an expected value of 143, giving a critical ratio of 30.52 and indicating departure from multivariate normality. All confidence intervals reported below are therefore bias-corrected bootstrap intervals, which do not rely on the normality assumption. Mahalanobis distance identified 21 cases exceeding the χ² criterion at p < 0.001 (maximum D² = 58.74). These cases were retained, since none represented impossible or out-of-range responses, and their influence was evaluated directly in the robustness analysis reported below.

### Common method variance

In Harman’s single-factor test the first unrotated component accounted for 34.41% of the total variance, below the 50% threshold, and 14 components had eigenvalues exceeding one. A single-factor confirmatory model in which all indicators loaded on one latent variable fitted the data far worse than the hypothesised measurement model (χ² = 805.22, df = 44, χ²/df = 18.30, CFI = 0.876, RMSEA = 0.167 versus χ² = 40.64, df = 41, χ²/df = 0.99, CFI = 1.000, RMSEA = 0.000; Table 1). Common method variance is therefore unlikely to account for the observed relationships, although it cannot be eliminated given the self-report design.

**Table 1:** Fit indices for the measurement and structural models.

Model	χ²	df	χ²/df	CFI	TLI	RMSEA [90% CI]	SRMR
Communication Skills Scale (second-order, 25 items)	939.46	271	3.47	0.880	0.867	0.063 [0.059, 0.067]	0.052
Entrepreneurship Scale (one factor, 36 items)	2155.11	594	3.63	0.863	0.854	0.065 [0.062, 0.068]	0.045
Career Awareness Scale (second-order, 18 items)	532.09	131	4.06	0.924	0.911	0.070 [0.064, 0.076]	0.045
Pooled measurement model	40.64	41	0.99	1.000	1.000	0.000 [0.000, 0.027]	0.013
Structural model (hypothesised)	40.64	41	0.99	1.000	1.000	0.000 [0.000, 0.027]	0.013
Total-effect model (mediator omitted)	9.00	13	0.69	1.000	1.000	0.000 [0.000, 0.027]	0.007
Single-factor model (common method variance test)	805.22	44	18.30	0.876	0.845	0.167 [0.157, 0.177]	0.072
Alternative (reversed) structural model	40.64	41	0.99	1.000	1.000	0.000 [0.000, 0.027]	0.013

*Note.* N = 622. χ²/df = chi-square to degrees of freedom ratio; CFI = comparative fit index; TLI = Tucker–Lewis index; RMSEA = root mean square error of approximation; SRMR = standardised root mean square residual. Acceptable fit: χ²/df < 5, CFI ≥ 0.90, TLI ≥ 0.90, RMSEA ≤ 0.08, SRMR ≤ 0.08. The structural and reversed models are statistically equivalent reparameterisations of the pooled measurement model and therefore share its fit.

### Measurement model

Item-level confirmatory factor analyses of the three instruments yielded acceptable fit on the residual-based and parsimony-adjusted indices, with χ²/df between 3.47 and 4.06, RMSEA between 0.063 and 0.070 and SRMR between 0.045 and 0.052, although the incremental indices were more modest for the Communication Skills Scale (CFI = 0.880) and the Entrepreneurship Scale (CFI = 0.863) than for the Career Awareness Scale (CFI = 0.924); all fit statistics are reported in Table 1. Because the incremental indices for two instruments fell below the conventional 0.90 threshold at item level, we report them transparently here rather than only at the aggregated level, and return to this in the limitations.

The pooled measurement model, containing all three constructs simultaneously with communication skills and career awareness represented by their four dimension scores and entrepreneurial tendencies by three item parcels, fitted the data very well (χ² = 40.64, df = 41, χ²/df = 0.99, CFI = 1.000, TLI = 1.000, RMSEA = 0.000, 90% CI [0.000, 0.027], SRMR = 0.013). All standardised loadings were significant at p < 0.001 and ranged from 0.731 to 0.953 (Table 2). Composite reliability ranged from 0.885 to 0.965 and average variance extracted from 0.660 to 0.903, satisfying conventional criteria for convergent validity.

**Table 2:** Pooled measurement model: Factor loadings, reliability and convergent validity.

Construct	Indicator	λ	α	CR	AVE
Communication skills	Communication principles and basic skills	0.833	0.807	0.885	0.660
	Self-expression	0.793	0.756		
	Active listening and non-verbal communication	0.884	0.811		
	Willingness to communicate	0.731	0.703		
	Total scale (25 items)	—	0.918		
Career awareness	Professional development disposition	0.792	0.834	0.891	0.670
	Professional readiness	0.823	0.805		
	Occupational awareness	0.829	0.826		
	Occupational self-confidence	0.831	0.782		
	Total scale (18 items)	—	0.929		
Entrepreneurial tendencies	Parcel 1 (12 items)	0.953	0.883	0.965	0.903
	Parcel 2 (12 items)	0.951	0.889		
	Parcel 3 (12 items)	0.946	0.890		
	Total scale (36 items)	—	0.960		

*Note.* N = 622. λ = standardised factor loading, all p < 0.001; α = Cronbach’s alpha; CR = composite reliability; AVE = average variance extracted. Criteria: λ ≥ 0.50, α and CR ≥ 0.70, AVE ≥ 0.50.

Discriminant validity was examined in three complementary ways, given the magnitude of the associations involving entrepreneurial tendencies. First, all heterotrait–monotrait ratios fell below the 0.85 threshold: 0.707 for communication skills and career awareness, 0.822 for communication skills and entrepreneurial tendencies, and 0.807 for career awareness and entrepreneurial tendencies. Second, nested model comparisons in which each latent correlation was constrained to unity fitted significantly worse than the freely estimated model (Δχ²(3) = 835.44, 741.42 and 692.61 for the communication–career awareness, communication–entrepreneurship and career awareness–entrepreneurship pairs respectively, all p < 0.001), providing direct evidence against construct redundancy. Third, the Fornell–Larcker criterion was satisfied for the communication skills–career awareness pair (latent r = 0.732 against a smaller square root of AVE of 0.812) but not for the two pairs involving entrepreneurial tendencies, where latent correlations of 0.835 and 0.821 marginally exceeded the corresponding values of 0.812 and 0.819.

We report this divergence rather than selecting the criteria that favour the model. Simulation evidence indicates that the Fornell–Larcker criterion performs poorly at detecting a genuine lack of discriminant validity and that the heterotrait–monotrait ratio is the more sensitive test [52], and the nested comparisons provide direct evidence that the constructs are not redundant. We therefore treat the constructs as empirically distinguishable, while noting in the limitations that communication skills and entrepreneurial tendencies are tightly coupled in this population.

### Descriptive statistics and correlations

Means, standard deviations, distributional statistics and bivariate correlations are reported in Table 3. All three variables were positively and significantly intercorrelated. Communication skills correlated strongly with entrepreneurial tendencies (r = 0.772, p < 0.001) and moderately to strongly with career awareness (r = 0.651, p < 0.001), and career awareness correlated strongly with entrepreneurial tendencies (r = 0.760, p < 0.001).

**Table 3:** Descriptive statistics, correlations and discriminant validity.

Variable	1	2	3	M	SD	Skewness	Kurtosis
1. Communication skills	**0.812**			3.842	0.534	−0.258	−0.074
2. Career awareness	0.651**	**0.819**		3.798	0.608	−0.031	−0.271
3. Entrepreneurial tendencies	0.772**	0.760**	**0.950**	3.796	0.607	−0.053	−0.522

*Note.* N = 622. Values below the diagonal are Pearson correlations among observed composite scores; values on the diagonal (bold) are the square root of the average variance extracted from the pooled measurement model. Latent correlations were 0.732 (1–2), 0.835 (1–3) and 0.821 (2–3). Heterotrait–monotrait ratios were 0.707 (1–2), 0.822 (1–3) and 0.807 (2–3), all below 0.85. Means and standard deviations are reported to three decimal places because entrepreneurial tendencies and career awareness are indistinguishable at two. ** p < 0.01.

### Structural model and hypothesis tests

**Fig 2:**
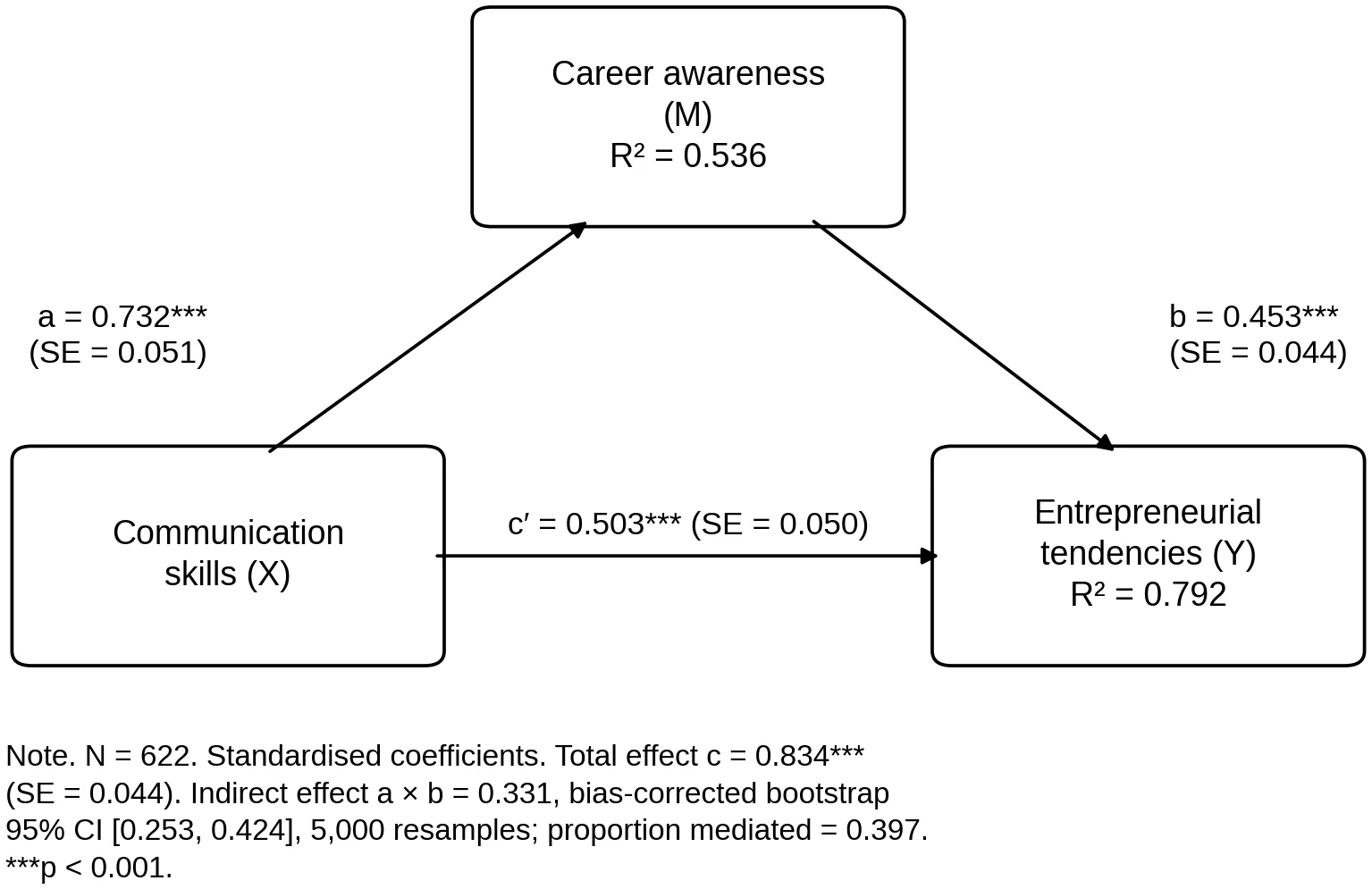
Standardised results of the mediation model. N = 622. Standardised coefficients are reported. Total effect c = 0.834 (SE = 0.044), p < 0.001. Indirect effect a × b = 0.331, bias-corrected bootstrap 95% CI [0.253, 0.424], 5,000 resamples; proportion mediated = 0.397. *** p < 0.001.

The structural model fitted the data well (Table 1). Standardised estimates are presented in Fig 2 and Table 4.

**Table 4:** Structural model and mediation results.

Path	B	SE	β	z	95% CI (β)
*Total effect*					
Communication skills → Entrepreneurial tendencies (c)	1.031	0.044	0.834	—	[0.796, 0.868]
*Direct effects*					
Communication skills → Career awareness (a)	0.838	0.051	0.732	16.47	[0.659, 0.791]
Career awareness → Entrepreneurial tendencies (b)	0.488	0.044	0.453	11.08	[0.354, 0.560]
Communication skills → Entrepreneurial tendencies (c′)	0.621	0.050	0.503	12.40	[0.393, 0.602]
*Indirect effect*					
Communication skills → Career awareness → Entrepreneurial tendencies (a × b)	—	—	0.331	—	[0.253, 0.424]
*Explained variance and effect share*					
Career awareness (R²)			0.536		
Entrepreneurial tendencies (R²)			0.792		
Proportion of total effect mediated			0.397		

*Note.* N = 622. B = unstandardised coefficient; β = standardised coefficient. All direct paths significant at p < 0.001. Confidence intervals are bias-corrected bootstrap intervals based on 5,000 resamples; the percentile interval for the indirect effect was [0.252, 0.422]. An interval excluding zero indicates statistical significance.

The causal steps were as follows. The total effect of communication skills on entrepreneurial tendencies, estimated without the mediator, was positive and significant (c = 0.834, SE = 0.044, p < 0.001). Communication skills predicted career awareness (a = 0.732, SE = 0.051, z = 16.47, p < 0.001), supporting H1, and accounted for 53.6% of its variance. Career awareness predicted entrepreneurial tendencies with communication skills controlled (b = 0.453, SE = 0.044, z = 11.08, p < 0.001), supporting H2. The direct effect of communication skills on entrepreneurial tendencies remained positive and significant with career awareness in the model (c′ = 0.503, SE = 0.050, z = 12.40, p < 0.001), supporting H3. Together, the two predictors explained 79.2% of the variance in entrepreneurial tendencies.

### Mediation

The indirect effect of communication skills on entrepreneurial tendencies through career awareness was 0.331, with a bias-corrected bootstrap 95% confidence interval of [0.253, 0.424] based on 5,000 resamples; the percentile interval was almost identical, [0.252, 0.422]. Because the interval excluded zero, H4 was supported. The indirect effect accounted for 39.7% of the total effect, indicating that a substantial but minority share of the relationship operates through career awareness, with the remainder transmitted directly. As the direct effect remained significant with the mediator included, the pattern corresponds to partial rather than complete mediation; this descriptive label depends, however, on the statistical power available to detect the residual direct effect and should not be read as evidence for additional unmodelled mechanisms [54].

### Robustness

Two checks were conducted. First, an alternative model was estimated in which career awareness predicted communication skills and communication skills mediated the effect on entrepreneurial tendencies. This model produced identical fit to the hypothesised model (χ² = 40.64, df = 41; Table 1), which is expected because the two specifications are statistically equivalent in a cross-sectional design. The hypothesised ordering is therefore justified on theoretical rather than empirical grounds, a limitation addressed below. Second, the structural model was re-estimated with the 21 multivariate outliers excluded (N = 601). No path coefficient changed by more than 0.022 (a = 0.750, b = 0.431, c′ = 0.520, indirect effect = 0.323), and every inferential conclusion was unchanged, indicating that the results do not depend on the retention of these cases.

## Discussion

The purpose of this study was to examine the relationship between communication skills and entrepreneurial tendencies among prospective sport managers and to test whether career awareness mediates that relationship within the framework of social cognitive career theory. All hypothesised relationships were supported, and the results provide evidence for a partial mediating effect of career awareness. Communication skills therefore appear to contribute to entrepreneurial tendencies both directly and indirectly, by strengthening students’ understanding of career opportunities and requirements.

### Communication skills predict career awareness (H1)

Communication skills positively predicted career awareness (β = 0.73, p < 0.001), accounting for over half of its variance. Students with stronger interpersonal communication competence appear more capable of recognising career-related opportunities, understanding professional requirements and engaging in career planning. From an SCCT perspective, communication competence supports the adaptive learning experiences that shape career-related beliefs and behaviours [9,10]. In sport management, where interaction with athletes, coaches, staff, sponsors and multiple stakeholders is central, communication skills plausibly function as a personal resource that facilitates career development and informed decision-making.

Communication competence may also reflect a broader transferable skill set closely linked to socio-emotional capabilities that matter for academic adjustment and professional functioning; consistent with this, communication skills have been found to predict social-emotional competence [13]. Universities can moreover foster communication competence through structured, practice-oriented educational provision, which indicates that it is a developable and context-relevant competency rather than a fixed trait [56].

### Career awareness predicts entrepreneurial tendencies (H2)

Career awareness positively predicted entrepreneurial tendencies (β = 0.45, p < 0.001). Students who are more aware of career pathways and professional expectations appear more likely to display entrepreneurial characteristics such as initiative, opportunity recognition and proactive career behaviour. In sport management education, career awareness may help students identify alternative pathways — sport event management, fitness entrepreneurship, sport marketing and consultancy among them — and thereby increase their readiness to pursue innovative and self-directed career strategies.

This finding is consistent with literature linking career awareness to psychological resources in career development: greater awareness has been associated with career self-efficacy, such that students with a clearer view of the occupational field report more confidence in evaluating opportunities and making career decisions [8]. Relatedly, sport students face psychosocial and structural barriers in shaping their professional trajectories, and career awareness may support adaptive coping in response to such constraints [57].

### Communication skills predict entrepreneurial tendencies (H3)

Consistent with the proposed model, communication skills had a positive effect on entrepreneurial tendencies with career awareness controlled (β = 0.50, p < 0.001). Communication competence plausibly supports entrepreneurship-related outcomes by enabling students to build networks, collaborate, negotiate and mobilise resources — capabilities that are especially relevant within sport organisations. From an applied perspective, students with strong communication skills may be better equipped to develop sport-related projects, promote services and create solutions in dynamic sport settings. Supporting this relationship, communication skills have been reported to predict entrepreneurship and to explain a substantial proportion of variance in entrepreneurial tendencies among sport management students [6], and research on entrepreneurship education in sport-specific learning contexts has emphasised the role of interpersonal capability in developing entrepreneurial psychology among sport majors [29,30].

### The mediating role of career awareness (H4)

The principal contribution of this study is the finding that career awareness partially mediated the relationship between communication skills and entrepreneurial tendencies (indirect effect = 0.331, 95% CI [0.253, 0.424]). Communication skills therefore appear to foster entrepreneurial tendencies not only directly but also indirectly, by strengthening students’ awareness of career opportunities and professional requirements. Students who communicate effectively may be better able to translate interpersonal competence into entrepreneurial behaviour when they also hold a clearer understanding of career development processes and available pathways.

This mediation effect provides empirical support for SCCT-based accounts in which career outcomes emerge through interaction among individual competencies, contextual processes and personal agency [9,10]. The plausibility of communication operating as a transmitting mechanism has also been established in sport-related contexts: communication skills have been found to mediate the relationship between empathy, team cohesion and competition performance, demonstrating that communication can link psychosocial resources to meaningful outcomes [31]. Extending this perspective to prospective sport managers, communication skills may strengthen entrepreneurial tendencies partly by improving students’ capacity to engage with career-related environments, identify opportunities and act proactively.

The magnitude of the mediated pathway is as informative as its significance. Career awareness carried 39.7% of the total effect of communication skills on entrepreneurial tendencies, which locates the mechanism precisely: it is substantial enough that models omitting it are meaningfully incomplete, yet it leaves the majority of the effect operating directly. This pattern is theoretically coherent rather than anomalous. Communication skill plausibly contributes to entrepreneurial tendency along two distinct routes — informationally, by enlarging what a student learns about the occupational field, and instrumentally, because entrepreneurial activity is itself conducted through persuasion, negotiation and representation. Career awareness captures the first route; the residual direct effect is consistent with the second. The finding therefore does not indicate a partially specified model so much as a competence that operates through more than one channel, only one of which is cognitive.

### Interpreting the explained variance

The proportion of variance explained in entrepreneurial tendencies (79.2%) is high by the standards of research in this area and warrants comment. Three considerations bear on its interpretation. First, the estimate derives from a latent-variable model in which relationships are corrected for measurement error, and will therefore exceed the corresponding observed-variable estimate; the analogous figure computed from composite scores in these data is 71%. Second, all three constructs were measured by self-report at a single time point, and while common method variance was assessed by two independent procedures and found unlikely to account for the observed relationships, it cannot be eliminated as a contributor. Third, and most substantively, there is conceptual proximity between the predictor and the criterion: the entrepreneurship measure encompasses leadership and self-efficacy components, and items indexing these characteristics may partly overlap with interpersonal competence. This proximity is visible in the psychometric results, where the latent correlation of 0.835 between communication skills and entrepreneurial tendencies satisfied the heterotrait–monotrait criterion and the nested comparison but not the Fornell–Larcker criterion. The estimate is therefore better read as evidence that these competencies are tightly coupled in this population than as an indication that entrepreneurial tendency is nearly fully determined by the two predictors examined here.

## Implications

### Theoretical implications

The contribution of this study is not the demonstration that three positively correlated constructs are positively correlated, but an account of why communication competence bears on entrepreneurial disposition and a quantification of how much of that relationship is cognitively mediated. A theoretical contribution consists in explanatory logic rather than in the accumulation of supported hypotheses [58,59], and the present findings advance such logic in three respects.

First, they move social cognitive career theory in sport management education from a descriptive framing to a tested mediational specification. SCCT is frequently invoked in this literature as a rationale for expecting competencies to matter, but its central mechanism — that competencies act on career outcomes through cognitive appraisals rather than directly — is rarely modelled, with the consequence that the theory is used to motivate direct-effect studies that cannot in principle test it. By specifying and estimating the mediational structure the theory implies, this study supplies evidence for the mechanism rather than for the prediction alone. The finding that the mediated pathway is substantial but partial is itself theoretically consequential: it indicates that SCCT’s cognitive route is one of at least two through which interpersonal competence operates, and that a purely cognitive reading of the theory would be incomplete in domains where the competence in question is also instrumentally required for the outcome behaviour.

Second, the study identifies which cognitive construct carries the effect. SCCT specifies a family of appraisals — self-efficacy beliefs, outcome expectations, occupational knowledge — and applied work has tended to treat these interchangeably or to select among them without justification. Career awareness is distinctive within this family because it concerns knowledge of the occupational world rather than beliefs about the self, and the results indicate that this externally directed appraisal transmits a meaningful share of the effect. This suggests a refinement to the theory’s application in vocationally oriented fields: where occupational structures are informal and poorly documented, world-of-work knowledge may be the binding constraint on career-relevant action, and self-referential appraisals may matter less than the literature’s emphasis on self-efficacy would imply.

Third, the study specifies a mechanism whose operation should be context-dependent in a testable way. Our account holds that communication skill matters for career awareness because much of what students need to know about sport management careers is transmitted conversationally rather than documented. If that account is correct, the strength of the a path is a function of how informally a field’s opportunity structures are organised, and should therefore be weaker in occupational domains with codified entry pathways. This is a prediction about boundary conditions rather than a claim of universality, and it locates the Turkish sport management context as theoretically informative in its own right rather than as an incidental site of replication [60].

### Managerial and policy implications

The results carry a specific implication for curriculum design that follows from the size of the mediated pathway rather than from its significance. Because approximately 40% of the effect of communication skills on entrepreneurial tendencies operates through career awareness, a programme that strengthens students’ communication competence without also developing their understanding of the occupational field forgoes a substantial share of the available benefit. Communication training and career provision are frequently administered separately — the former as coursework, the latter as a support service accessed on student initiative — and these findings indicate that the separation is costly. Four recommendations follow.

First, communication instruction should be coupled to career-information tasks rather than taught as a generic competence. The mechanism identified here is that communicatively skilled students extract more career-relevant information from their environment; curricula can operationalise this directly by making information-gathering the assessed output of communication coursework. Structured informational-interviewing assignments, in which students identify practitioners in a sport subsector, secure and conduct an interview, and report what they learned about entry pathways and unmet needs in that subsector, exercise the communication skill and build career awareness simultaneously. This is a low-cost redesign of existing provision rather than an addition to it.

Second, career-awareness provision should be positioned early in the programme. Career awareness functions as a precursor to other components of career development rather than a late-stage outcome, and the mediational logic implies that its absence in the early years limits the return on communication instruction delivered at the same time. Programmes that concentrate career activity in the final year invert this sequence.

Third, career awareness should be treated as an assessable programme learning outcome, and measured. The instrument used here is short, freely available and validated for sport sciences students, which makes periodic administration feasible. Its diagnostic use is more valuable than its evaluative use: students who score high on communication skills but low on career awareness constitute the group with the largest unrealised entrepreneurial potential, since the competence is present but the knowledge that would direct it is not. This group is invisible to conventional academic performance monitoring and can be identified at negligible cost.

Fourth, at the policy level, accreditation and programme-approval frameworks for sport management education should require evidence of structured industry engagement rather than of career-service availability. The distinction matters because the mechanism operates through interaction with practitioners, which institutional provision can enable but cannot substitute for. Formal partnerships with federations, clubs and sport enterprises that guarantee students recurring practitioner contact — rather than a single internship placement — are the provision most closely aligned with the process documented here. For programmes in rapidly expanding systems, where graduate supply outpaces the growth of formal managerial appointments, this alignment has direct employability consequences: much of the sector’s employment growth lies in self-initiated activity, and entrepreneurial capacity is accordingly a component of employability rather than an alternative to it.

### Limitations and directions for future research

Five limitations qualify these conclusions, and each indicates a specific line of subsequent enquiry.

First, the design is cross-sectional, which is the most consequential limitation because the hypothesised ordering is not empirically identified: as the robustness analysis confirmed, a model reversing communication skills and career awareness produced identical fit, and the ordering rests on theoretical argument alone. Resolving this requires temporal design, and specifically a cross-lagged panel study spanning at least three annual waves, which would establish whether change in communication skills precedes change in career awareness or the reverse — and would also permit the more interesting possibility that the relationship is reciprocal, with career awareness motivating further communicative engagement. A stronger test still is experimental: randomly assigning students to a career-awareness intervention while holding communication instruction constant would establish whether manipulating the mediator alters entrepreneurial tendency, which is the causal claim on which the practical recommendations above ultimately depend. We regard this as the single most valuable next study, because the managerial implication of a mediated pathway is only as secure as the causal status of the mediator.

Second, communication skills and entrepreneurial tendencies were empirically tightly coupled, with a latent correlation of 0.835. The constructs satisfied the heterotrait–monotrait criterion and were clearly distinguishable in nested model comparisons, but they did not satisfy the more conservative Fornell–Larcker criterion, and the entrepreneurship instrument includes leadership and self-efficacy content that may overlap conceptually with interpersonal competence. Research using an entrepreneurship measure with narrower content coverage, or behavioural rather than dispositional indicators, would establish how much of the direct effect reported here reflects a substantive relationship as opposed to construct overlap.

Third, all constructs were measured by self-report, and entrepreneurial tendency is a disposition rather than a behaviour. Dispositions and actions diverge, and the practically consequential question is whether students high in entrepreneurial tendency subsequently create ventures. Research following sport management graduates into the labour market and recording actual entrepreneurial activity would establish whether the mechanism documented here extends to behaviour, and would additionally allow observer-rated or performance-based assessment of communication skill, breaking the shared-method dependence of the present estimates.

Fourth, career awareness was examined as the sole mediator, and the mediated pathway was partial. Competing cognitive candidates — career self-efficacy, career adaptability, perceived employability — are theoretically plausible and have been examined separately in adjacent literatures. The informative study is not one that adds another mediator in isolation but one that enters them simultaneously, since this would determine whether career awareness retains its effect when self-referential appraisals are controlled. Our theoretical argument predicts that it will, and that world-of-work knowledge will dominate self-efficacy in this field; a multiple-mediator design would adjudicate that prediction.

Fifth, the sample was drawn from a single national context and a single field, and the boundary condition proposed above is untested. Because our account attributes the communication–career awareness link to the informality of sport’s opportunity structures, the mechanism should attenuate in occupational fields with codified entry pathways. A comparative study contrasting sport management with a professionally regulated field such as nursing or accountancy within the same educational system, or contrasting sport management across systems differing in labour-market formalisation, would test this directly. Such a study would generate knowledge that neither context yields alone: it would establish whether communication skill is a general antecedent of career awareness or a compensatory resource that matters most where institutional information provision is weakest.

Three measurement and procedural constraints should also be noted. The incremental fit indices for the Communication Skills Scale and the Entrepreneurship Scale fell below the conventional 0.90 threshold at item level, indicating that these instruments’ item-level structures are not reproduced perfectly in this population even though the residual-based indices were acceptable and the aggregated measurement model fitted well; future work in Turkish sport science samples might usefully revisit their factor structures. The near-perfect fit of the pooled model reflects in part the aggregation of items into dimension scores and parcels, which reduces the number of testable covariance constraints, and the item-level results are reported alongside it for this reason. Finally, a response rate could not be calculated under the recruitment procedure used, since the number of students who received but did not open the invitation is unknown; the sample was also unevenly distributed across the 16 contributing institutions, so institution-level clustering could not be modelled with adequate precision for the smaller sites; and the internal consistency of the 36-item entrepreneurship measure (α = 0.960) is high enough to suggest some item redundancy.

## Conclusions

Among prospective sport managers, communication skills are associated with entrepreneurial tendencies both directly and through career awareness, which transmits approximately two-fifths of the total effect. The substantive point is not that these competencies covary but that a determinate and measurable share of the relationship depends on students possessing an articulated understanding of the occupational field in which their communicative competence might be exercised. Interpersonal skill, on this evidence, is a resource whose entrepreneurial value is partly contingent on knowledge of where to direct it. For sport management education, the implication is that communication instruction and career provision are complements rather than parallel offerings, and that programmes treating them as separate functions realise only part of the benefit that either could deliver.

## Supporting information

S1 Script Reproducible analysis script. Python script reproducing all reported statistics from the raw data file, including sample screening, reliability, assumption tests, common method variance tests, all confirmatory factor models, discriminant validity tests, the structural model and the bootstrap mediation analysis.
(PY)
